# Can the word superiority effect be modulated by serial position and prosodic structure?

**DOI:** 10.3389/fpsyg.2022.915666

**Published:** 2022-08-05

**Authors:** Yousri Marzouki, Sara Abdulaziz Al-Otaibi, Muneera Tariq Al-Tamimi, Ali Idrissi

**Affiliations:** ^1^Department of Social Sciences, College of Arts and Sciences, Qatar University, Doha, Qatar; ^2^Department of English Literature and Linguistics, College of Arts and Sciences, Qatar University, Doha, Qatar

**Keywords:** word superiority effect, prosodic structure, serial position function, orthography, nonconcatenative morphology, Arabic

## Abstract

In this study, we examined the word superiority effect in Arabic and English, two languages with significantly different morphological and writing systems. Thirty-two Arabic–English bilingual speakers performed a post-cued letter-in-string identification task in words, pseudo-words, and non-words. The results established the presence of the word superiority effect in Arabic and a robust effect of context in both languages. However, they revealed that, compared to the non-word context, word and pseudo-word contexts facilitated letter identification more in Arabic than in English. In addition, the difference between word and pseudo-word contexts was smaller in Arabic compared to English. Finally, there was a consistent first-letter advantage in English regardless of the context, while this was more consistent only in the word and pseudo-word contexts in Arabic. We discuss these results in light of previous findings and argue that the differences between the patterns reported for Arabic and English are due to the qualitative difference between word morphophonological representations in the two languages.

## Introduction

The ability to read is a complex skill that minimally requires the ability to identify words and recognize their constituent orthographic units (Chanceaux and Grainger, 2012). The cognitive mechanisms involved in single-letter identification within words are paramount to attaining the necessary high level of automaticity in reading (Marzouki and Grainger, 2014). Related to this complex skill is the so-called word “superiority effect” phenomenon, first reported by Reicher (1969) and Wheeler (1970). These authors show that a letter embedded in a word was identified more accurately than the same letter embedded in a pseudo-word or non-word. For example, the accuracy with which the letter M is identified in the word MOVIE would be significantly higher than when the same letter appears in the pseudo-word MAVIE or the non-word MAPVA. This effect follows from the general capacity of humans to be better at identifying and processing whole words than isolated letters or non-words (Starrfelt et al., 2013; Sand et al., 2016).

Many studies have shown the word superiority effect to be robust under various experimental conditions such as those including noise or presentation speed (Spector and Purcell, 1977; Johnston, 1981). In Reicher's (1969) study, participants were presented with either four-letter words, four-letter non-words, or a single letter. For example, the word “WORD” was shown on the screen followed by a mask accompanied by two single letters as suggested response choices. Participants performed a two-alternative forced-choice task in which they must choose between the letters “D” (found in the target WORD) and “K” (lacking in the target WORD). The results revealed that the participants responded more accurately in the word context than in the non-word context. Interestingly, this study also shows that the accuracy with which a letter is identified is higher when the latter is embedded in an orthographically legal string of letters, that is, in words and pseudo-words than when it appears in orthographically illegal strings of letters, that is, in non-words. Similarly, Wheeler (1970) further confirmed the robustness of the word superiority effect by using what has become to be known as the “Reicher–Wheeler” task, which differs from Reicher's (1969) initial experiment in that it controls for the serial position, the word-probe delay, and word frequency. The word superiority effect has been often interpreted as strong evidence of the presence of top-down modulation originating from the mental lexicon of the lower levels of visual word form recognition (Marchetti and Mewhort, 1986).

Hung et al. (1999) used the Reicher–Wheeler task to study the word superiority effect in Chinese, a language whose orthography is characterized by the inconsistency between the roles played by individual characters and words as units of perception during word reading. The authors reported results showing that words were more salient than characters as perception units in Chinese. Moreover, monomorphic words were recognized more accurately than bimorphemic words. These findings provide further evidence for top-down modulation during visual word identification.

In a study combining the Reicher–Wheeler task and ERP recording, Martin et al. (2006) found that letter identification in the word context was significantly more accurate and faster than in the non-word context. These authors also reported that the early ERP component, N1, was significantly modulated by the lexical status of the stimulus. Their results were taken to underscore the role played by visual word form representation in constraining letter identification at the very early prelexical stage.

In a study using the same technique as Martin et al.'s, Coch and Mitra (2010) specifically investigated the timecourse of word and pseudo-word superiority. These authors included a condition, the letter-in-X condition, where the target letter is embedded in a string of *X*s (e.g., B in XXBXX). They found that letter identification was more accurate in the word and pseudo-word conditions than in the non-word and letter-in-Xs conditions. Interestingly, their ERP results confirmed the presence of an early P150 and a late N400 component that could be associated with the word superiority effect.

In another study, Houpt et al. (2014) replicated the word superiority effect using words, pseudo-words, non-words, and Katakana character strings. The results show that participants responded more accurately in the context of words and pseudo-words than in the context of non-words and Katakana strings, both of which are more likely to activate top-down processing.

In light of the above, one may still ask whether bottom-up processing is involved at all in the word superiority effect. Recent accounts have shown that our ability to identify letters within strings can be attributed to some low-level visual factors, namely visual acuity, and crowding. These two factors were shown to provide the best explanation to date of the famous W-shaped curve, a specific case of the serial position function often observed in experimental settings involving stimuli composed of five-letter words. The W-shaped curve reflects a high level of accuracy in reporting letters in word-initial, word-medial, and word-final positions and significantly low accuracy in the medial non-fixed positions. The higher accuracy in identifying letters found in the outer positions in the letter string is explained by the reduced visual crowding that characterizes these positions. In fact, word-initial and word-final letters are, respectively, followed or preceded by only one neighboring element: on the right for word-initial letters and the left for word-final ones (Marzouki and Grainger, 2014; Grainger et al., 2016; Schubert et al., 2018).

**Figure 1:**
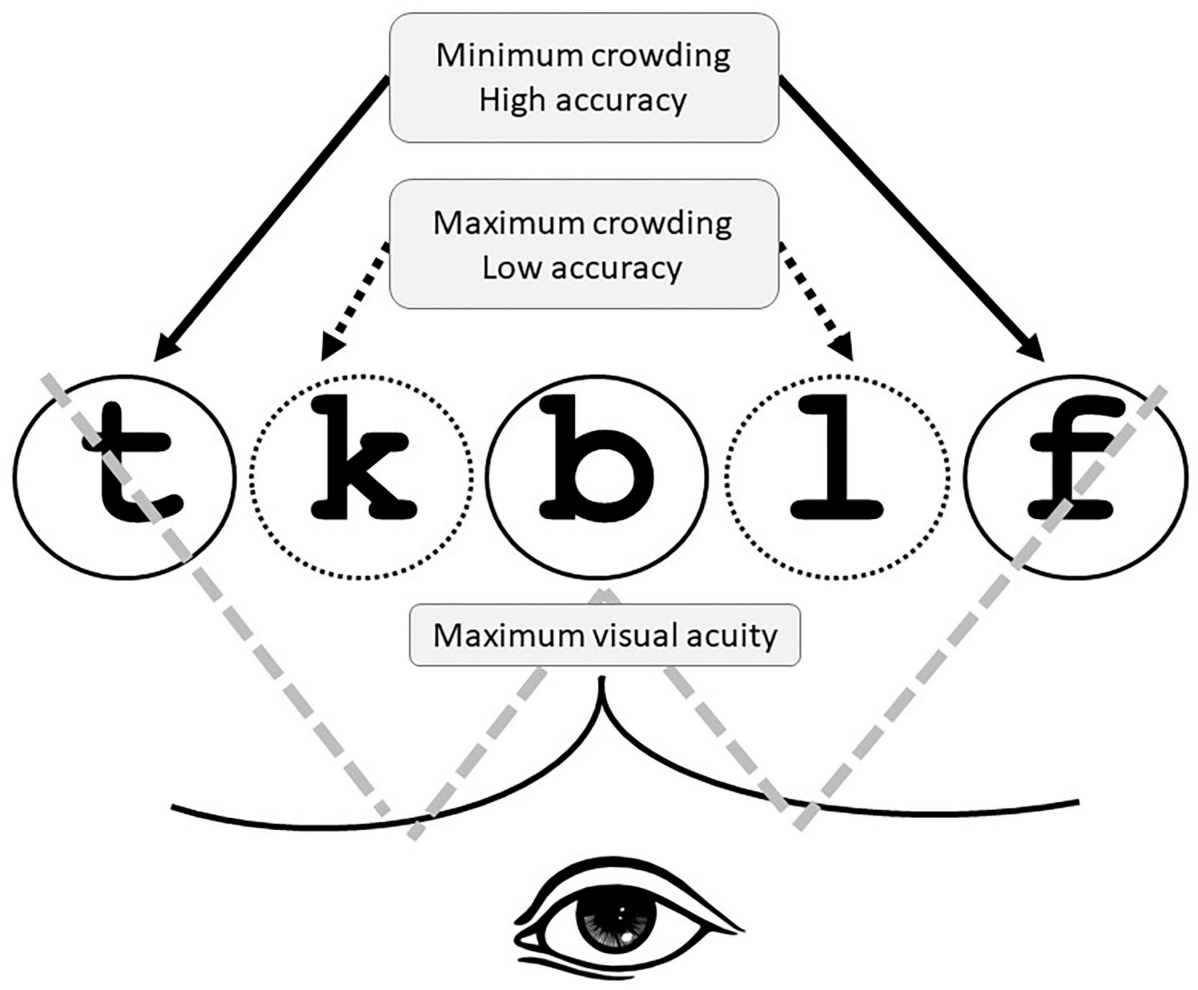
Illustration of the serial position function in the identification of the letters within the string of characters.

The ubiquity of visual crowding goes beyond lab experiments; it is considered a real-life phenomenon stemming from the inherent difficulty of perceiving or identifying a visual target in the context of neighboring flankers (Whitney and Levi, 2011; Yong et al., 2016; Ronconi and Bellacosa Marotti, 2017). Marzouki and Grainger (2014) experimentally manipulated the factors of crowding and visual acuity to directly test Tydgat and Grainger's (2009) proposal that the outer-letter effect is driven by visual crowding differences, whereas the central-letter effect is driven by visual acuity differences. They manipulated the stimulus duration as a way to manipulate visual crowding in the bottom-up input from the stimulus while keeping a fixed level of stimulus contrast. They also manipulated the distance separating the letters in strings. These authors found that the longer the stimulus presentation, the higher the accuracy, with the presence of a systematic outer-letter advantage throughout the whole range of durations. However, they also observed that the central-letter advantage increased with long exposures. Regarding the manipulation of the distance between letters in strings, they found that the larger this distance, the weaker the final-letter and the first-letter advantages, with the central-letter advantage being still higher in the fixated central letter. These findings were taken by Grainger et al. (2016) to confirm the hypothesis that the typical W-shaped serial position function for letter-in-string identification accuracy is likely to be the consequence of the combination of acuity and crowding effects as shown in Figure 1.

Three main mechanisms have been explored to explain the W-shaped phenomenon. First, visual acuity may account for the central-letter effect. Second, crowding can explain participants' higher accuracy in detecting letters with fewer neighbors (the word-initial and word-final letters). Third, spatial attention may play a role in orthographic processing. Thus, endogenous factors such as reading habits and directionality and exogenous factors such as the spatial cues and distribution of the letters operate conjointly with visual acuity and crowding to facilitate orthographic processing. Figure 1 shows how visual acuity and crowding contribute to the serial position function also referred to as a W-shaped curve that reflects the presence of a specialized system for parallel letter processing needed for the orthographic processing of information (see also Grainger et al., 2016).

### The present study

In a significant departure from the typical two-level design that contrasts two levels of context (i.e., the string type): words and non-words, we designed the current experiment in a way meant to allow us to dissociate the mechanisms associated with within-string letter identification from the mechanisms associated with the context. To achieve this, we manipulated pseudo-word and non-word stimuli (Jacobs and Grainger, 2005). We thus took into consideration the insight offered by Grainger and Jacobs (1994) dual read-out model, which predicts successful letter identification to be a function of the amount of noise in the context (e.g., the presence of pseudo-word superiority effect as a result of word misperception, see Grainger et al., 2003).

Arabic, a Semitic language characterized by its non-concatenative (or non-linear) morphological system, exhibits a unique word structure that allows for a systematic way to construct and structurally (phonologically and morphologically) distinguish between pseudo-words and non-words. It also offers a unique writing system that, unlike in English, overall mimics morphological structure.

In the Arabic morphological system, the components of a word, or morphemes, are typically intertwined with each other, unlike in languages such as English or Turkish, where morphemes (smaller units of form and meaning) are typically linearly attached one after the other. For example, the English word *restlessness* consists of the linear combination of three units identifiable through simple form-meaning correspondence (Nida, 1949): the root *rest*, bearing the lexical meaning and grammatical category of the noun {REST}, the adjectival suffix *-less*, carrying a privative meaning, something like {LACKING}, and the nominal suffix *-ness* meaning {STATE OF HAVING THE PROPERTIES OF THE ADJECTIVE}. Importantly, each one of these units is an integral, unbreakable continuous string of timing slots (C and V positions) (Halle and Vergnaud, 1980; Clements and Keyser, 1983) and melodic units (actual sounds) which can be represented as in (1).

**Table:** 

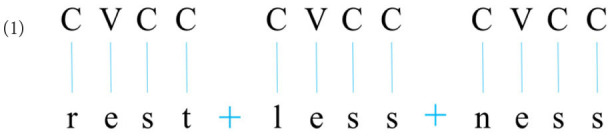

The sequencing of consonants and vowels in (1) is quasi-random. In other words, while it is CVCC for all three units, it need not be so for other units. In fact, it could be VCCVC (as in the root *active* [æktIv]) or VCC (as in the suffix *-ism* [Izm]) or CVC (as in the root *race* [res]), etc. The order of the C and V positions and the melodic elements associated with them is purely lexical, that is, it must be part of the lexical (learned) representation of each word. Moreover, this lexical order hardly changes: The sequencing of /r/, /e/, /s/, and /t/ in the root *rest* will typically always remain intact throughout the inflectional and derivational paradigms of this root (see *rested, rests, resting, unrest*, and *restful*). It is important to note that the only constraint on the sequencing of segments in English concerns some consonant combinations: Among the set of possible clusters, the attested ones always abide by specific phonotactic constraints of English. For example, while *rest* is permitted ^*^rset is not: being an impossible root initial cluster in English (Clements, 1990; Ladefoged, 1993).

Arabic shows a rather different pattern. In this language, unlike in (1), the morphemes of a word are typically not linearly ordered. Rather, they are intertwined with each other and can at best be represented as standing on parallel, independent layers, where each layer hosts a separate morpheme, as shown in (2) (McCarthy, 1979, 1981, 1989; Hammond, 1988; Yip, 1988; Idrissi, 1997, 2018).

**Table:** 

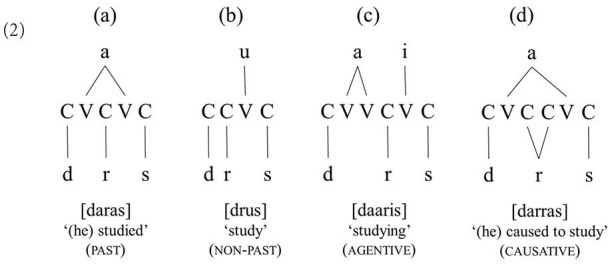

In this system, the core element of a word, its root, which carries the broad meaning of the word (e.g., STUDY), consists of consonants only and lies on a separate level from the vowels and the syllabic pattern, called the vowel melody and the template/word pattern, respectively. The vowel melody and template mainly convey grammatical properties (e.g., tense/aspect), but they may also often contribute semantic information (e.g., agentive and causative) (Idrissi, 2001; Arad, 2005).

Importantly, unlike its English counterpart, the root in Arabic emerges as a discontinuous unit, the constituent consonants of which may be non-adjacent but ordered predictably, namely as determined by the word template (CVCVC, CCVC, CVVCVC, and CVC_i_C_i_VC, where the index indicates that the medial consonants are identical). By definition, the word template exhibits a non-random, constant syllabic structure (Ussishkin, 2005). For example, while the past tense form of the simple past tense verb is *daras* “he studied” (word pattern CaCaC), the medial consonant is doubled (or geminated) in the past tense *and* causative form of the same verb: *darras* “he caused” (someone) to study (= he taught) (word pattern CaC_i_C_i_aC, with the medial Cs coindexed, i.e., identical). Similarly, while the CVCVC pattern, combined with the vowel melody {a}, indicates the past tense of the verb, the non-past is expressed with the CCVC pattern and the vowel melody {u} (i.e., CCuC). Thus, morphologically, unlike in English, words in Arabic consist of highly abstract units: a discontinuous consonantal root (e.g., *d-r-s*), a word syllabic pattern (e.g., simple active CVCVC and causative CVC_i_C_i_VC), and probably a vowel melody (active past *a-a* or active non-past *u*) (McCarthy, 1979, 1981).

It follows from this that, phonologically, English and Arabic show relatively different phonotactic patterns and prosodic structures. Essentially, in Arabic, the syllabic pattern, that is, the sequencing of consonants and vowels, is an essential part of the “identity” of a word. To wit, a word is a word in as much as it is built on a one-word pattern among a very limited set of word patterns existing in the language. This is not the case in English. Thus, while the sequencing of sounds is relatively arbitrary and is essentially lexical information in English, it is predictable and highly regular in Arabic. This makes the word pattern (also called template) a salient word unit in this language.

The difference between the English and Arabic morphological systems has significant implications for wordlikeness in the two languages. A real word in English is a word that does not violate the constraints on possible combinations of consonants (e.g., *blue* vs. ^*^lbue), while in Arabic, a real word is a form that coincides with attested and only attested root-word pattern combinations [e.g., the forms in (2)]. A pseudo-word would be a non-attested combination of an existing root and an existing word pattern, while a non-word would be the combination of a non-existing root and a non-existing word pattern. This has significant implications for what constitutes a “noisy” context in our experiment.

Another idiosyncratic feature of the Arabic language is its cursive and exclusively consonantal script (Elanwar et al., 2007). Unlike Latin alphabetic languages, Arabic written words present letters that represent only consonants and the three long vowels (short vowels are not indicated in everyday texts). In addition, these letters are mostly connected, that is, with no spaces between them. This feature imposes a new visual constraint that, to the best of our knowledge, was only directly tested for the first time by Jordan et al. (2010), who investigated the word superiority effect in Arabic. In their brief report, these authors used the two-alternative Reicher–Wheeler task in which the participants had to choose the appropriate stimulus that matches a previously and briefly presented stimulus among three choices: The word displayed simultaneously with a pseudo-word and a non-word both generated by scrambling the order of the letters in the word. Jordan et al.'s results revealed a word superiority effect with an advantage in accuracy for word over pseudo-words and for pseudo-words over non-words. However, the way their non-words were generated does not make them unpronounceable. In addition, the authors did not provide any information regarding the frequency and length of the word stimuli.

We used the Reicher–Wheeler paradigm to study the word superiority effect in Arabic while manipulating the serial position factor within a post-cued letter-in-string identification task. In addition, our stimuli offer us a way to indirectly test the potential effect of prosodic structure on the word superiority effect, given that our pseudo-words show existing word patterns while non-words do not.

Given the role morphological structure plays in word recognition (Hung et al., 1999) and the tight relationship between word prosodic structure, orthography, and morphology in Arabic (see discussion above), we hypothesize that (consonant) within-string letter identification in Arabic should be more sensitive to context (and more specifically to the prosodic structure of the context) than in English.

The aim of our study is 3-fold. First, we aim to investigate the word superiority effect in Arabic. We used bilingual readers with Arabic as the dominant language to establish a baseline condition against which we can assess the magnitude of the visual factors (visual acuity and crowding) mentioned above. Second, we test the extent of the interaction between morphological structure, prosodic structure, and the visual form of the word. In other words, we ask whether the serial position effect (a bottom-up feature) would modulate the word superiority effect (a top-down feature) in Arabic. Finally, we aim to replicate the findings of Jordan et al. (2010) by using more stringent criteria for the selection and inclusion of the experimental stimuli than in previous research. Our non-words were created by combining various consonantal root letters with random consonants in a way that leads to unpronounceable written stimuli that are as morphologically illegal as possible (see Appendix A).

## Methods

### Participants

A total sample of 32 Qatar University students aged from 22 to 35 years took part in the experiment for course credit. All participants were proficient in Arabic and English, with Arabic being their native language. They were all right-handed and reported having a normal or corrected-to-normal vision. The experiments were conducted at the NeuroCognition of Language Lab, and the experiment and participants' recruitment were approved by Qatar University Institutional Review Board.

### Stimuli and design

We used three types of stimuli for both Arabic and English: words, pseudo-words, and non-words (The complete list of stimuli can be acceded *via* this OSF link: https://mfr.osf.io/render?url=https://osf.io/8pt3w/?direct%26mode=render%26action=download%26mode=render). They were all composed of five letters. Sixty words were selected for each language. We excluded function words, surnames, and plurals. Words and pseudo-words in Arabic share the same word pattern. For example, the word 
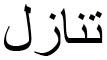
 [tanaazal] “to give-up” corresponds to the possible pseudo-words 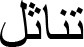 [tanaaθal], 
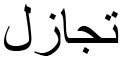
 [tažaazal], and 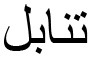 [tanaabal], while non-words are mere random unpronounceable sequences of five consonants, as in 
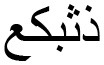
 [ðθbk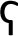].

**Figure 2:**
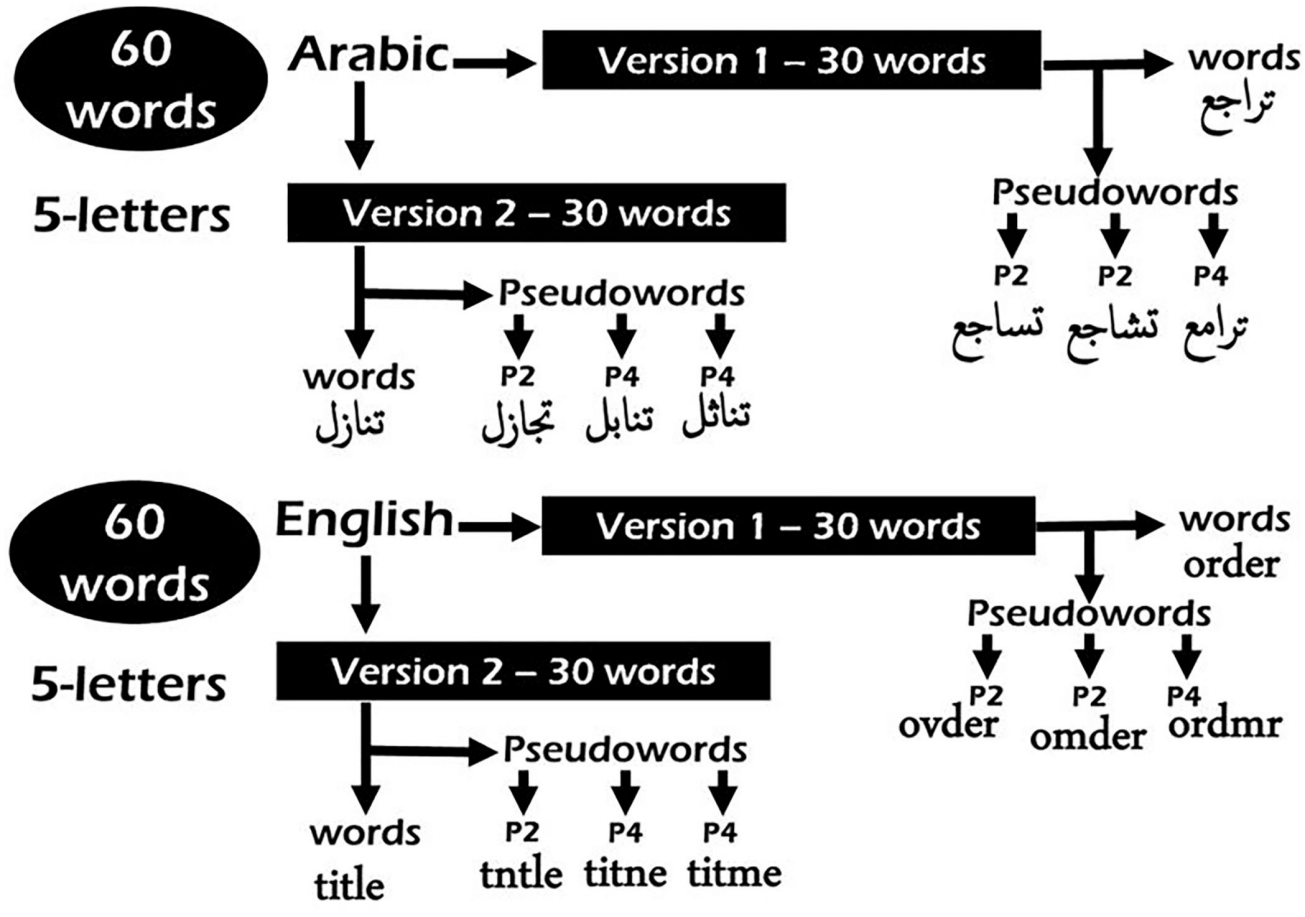
Examples of pseudo-words generated in Arabic and English from their corresponding word stimuli. See Appendix for the complete list of stimuli.

Word stimuli were assigned to three different lists with equivalent frequency for both languages. For Arabic, we used the type frequencies of roots and patterns as can be obtained from Aralex (Boudelaa and Marslen-Wilson, 2010). For English, we used Hyperspace Analog to Language (HAL) frequency norms (Lund and Burgess, 1996). Based on these metrics, we calculated the Zipf measure for the frequency per million words value for each list of words (Van Heuven et al., 2014). In English, the mean frequency counts for each list were as follows: List 1 = 4.06, List 2 = 4.03, and List 3 = 4.04; and in Arabic, they were as follows: List 1 = 4.53, List 2 = 4.54, and List 3 = 4.55. The sixty 5-letter words selected from the lists were split into two versions to be counterbalanced over participants following a standard Latin Square design. Each of the two versions includes thirty 5-letter words in Arabic and thirty 5-letter words in English. In each version, 60 pseudo-words were generated by changing either the second or fourth letter to maximize the chance for all consonants to be counterbalanced over these positions for all participants (see Figure 2 for examples of generated pseudo-words in both languages). The four pseudo-word lists (two for Arabic and two for English) were then evaluated by five judges. Three judges were native speakers of Arabic and rated the Arabic lists, and two were native speakers of English and rated the English lists. The judges were asked to decide how well the pseudo-word could be considered a plausible real word on a scale from 1 to 3. One means the farthest from being a real word and three the closest (see Appendix for the least plausible words retained for the experiment). Regarding the non-words, we created two lists of sixty non-pronounceable and meaningless five-letter non-words for both Arabic and English.

The experimental design is a 2 x 3 x 3 full factorial design with Language (Arabic vs. English), String Type (words vs. pseudo-words vs. non-words), and Letter Position (first vs. third vs. fifth letter) as within-participants factors. Response accuracy was collected as a dependent variable.

### Procedure

**Figure 3:**
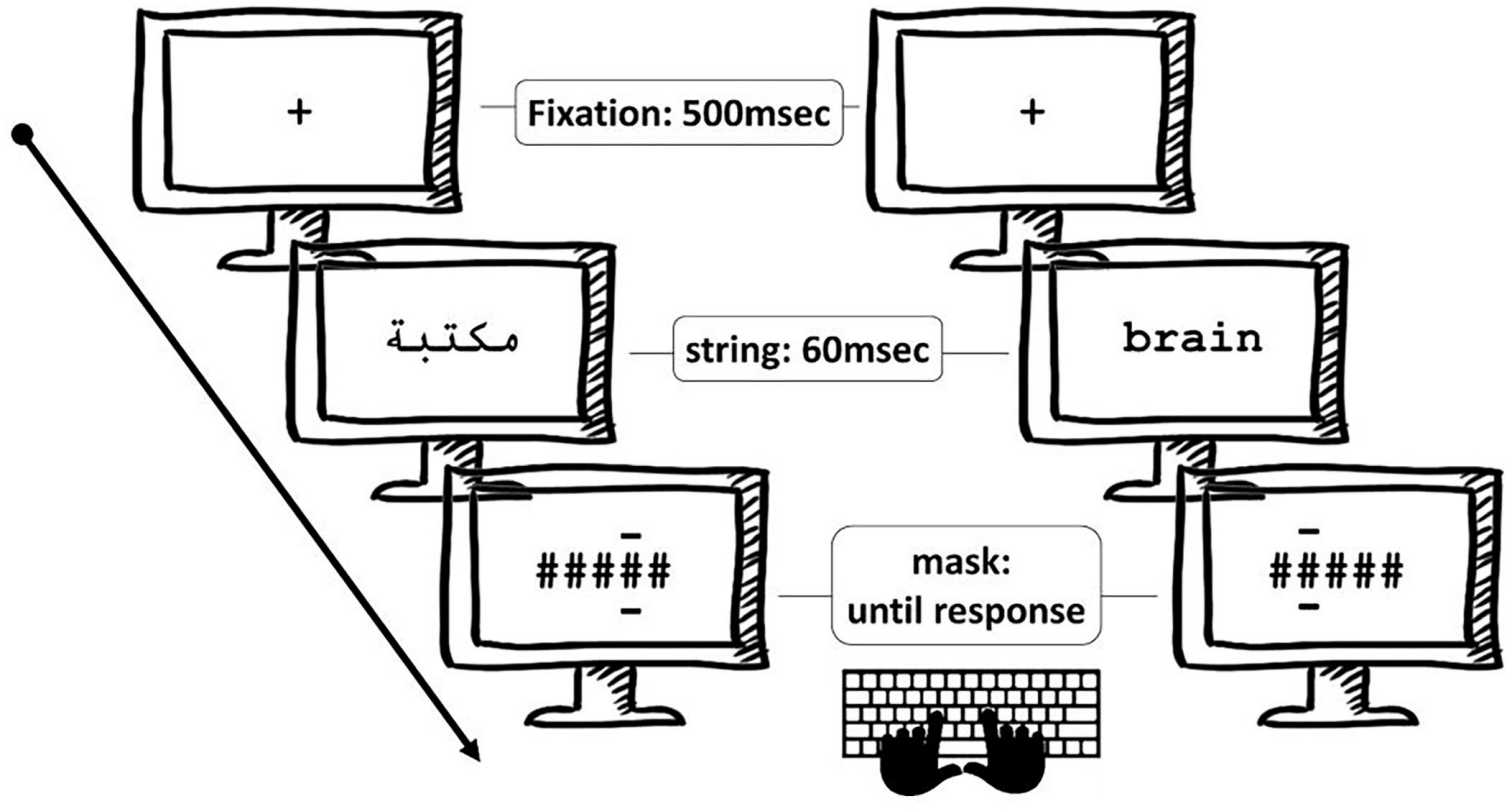
Illustration of the experimental procedure for the post-cued letter-in-string identification task with Arabic stimuli (left panel) and English stimuli (right panel). Letters in position 2 for both languages were post-cued in these two examples of trial.

The experiment was designed and administered using OpenSesame software (Mathôt et al., 2012). Participants were seated at an 80-cm distance in front of a computer screen on which stimuli were displayed in black on a white background in VGA mode (75-Hz refresh). Stimulus presentation proceeded as follows. First, a fixation point was shown for 500 msec. It was then immediately followed by a string of five-letter stimulus for 60 ms forming either a word, a pseudo-word, or a non-word. This string was then immediately masked with a string of hash marks with two horizontal lines serving as a post-cue indicating the position of the letter in the string that had to be reported. The mask remained until the participant responded using the dedicated buttons on the keyboard. The participants gave their responses by pressing the keyboard button corresponding to the letter that they thought was presented in the position indicated by the two dashes above and beneath it (see Figure 3).

A total of 180 stimuli (60 words/60 pseudo-words/60 non-words) were presented in a randomized block design. Each participant started with one bloc of stimuli from either Arabic or English with trials being presented randomly in each block. The total duration of the experiment is ~30 min, covering both the training and the main experimental sessions.

## Results

### Generalized linear mixed-effects modeling

We applied a logistic regression on accuracy data given the binary nature of the dependent variable (Y) with a value of 1 for the correct answer and 0 for the wrong identification of the target (data can be accessed *via* this OSF link: https://mfr.osf.io/render?url=https://osf.io/925ns/?direct%26mode=render%26action=download%26mode=render). The binary logistic regression was tested using a generalized linear mixed-effects model (GLME) with Language, String type, and Position as fixed effects and Participants and Items as random effects. The computation of the log-likelihood function for generalized linear mixed models was based on adaptive Gauss–Hermite quadrature as recommended by many authors (e.g., Kabaila and Ranathunga, 2019).

Three models were tested as shown in Table 1. The first model (M1) accounts for the baseline differences in language with no interaction between the fixed effects. This model is also referred to as the additive random intercept model. The second model (M2) is the random intercept model with interaction. The third model (M3) contains random intercepts but also random slopes allowing us to account for different slopes for the effect of language. In other words, each of the three models presented in Table 1 contains fixed effects for Language, Context, and Position. M1 is an additive model and does not account for interactions. M2 and M3 account for interactions but with different random effects. M2 contains a random intercept shared by all participants. M3, the retained model, has, in addition to a random intercept, a random slope in Language. This means that the rate at which individuals react to stimuli from Arabic or English differs from one participant to another. If an individual has a positive random effect, then they tend to be more accurate when exposed to Arabic stimuli than the average, while a negative random effect indicates that they are less accurate when exposed to Arabic than the average depending on the variance of the random effect of participants.

**Table 1:** Goodness-of-fit comparison between the three tested GLME models.

**Model specification**	**Df**	**AIC**	**BIC**	**Log Likelihood**	**Deviance**	**Chi square**	***p*-value**
M1: accuracy ~ language + context + position + (1 | participant) + (1 | items)	7	5438.7	5485.3	−2712.4	5424.7		-
M2: accuracy ~ language x context x position + (1 | participant) + (1 | items)	14	5415.7	5,509	−2693.9	5387.7	36.984^***^	0.0001
M3: accuracy ~ language x context x position + (1 + language | participant) + (1 | items)	18	5357.4	5477.2	−2660.7	5321.4	66.379^***^	0.0001

Significant effect is indicated by asterisk (***p < 0.001).

According to the goodness-of-fit statistics provided in Table 1, the optimal model is M3 with the least AIC and BIC values relative to M1 and M2 making it the most parsimonious model and with a significant reduction in deviance relative to the two previous ones.

### The optimal model parameters

The parameters of M3 are provided in Table 2 with the estimated values and their 95% confidence interval using the adjusted Wald method. The model reveals the presence of significant main effects of Language, String type, and Position (All *ps* < 0.0001). The two-way and the three-way interactions between these factors are also very significant (all *ps* < 0.0001). Although all *p*-values are highly significant (all *ps* < 0.0001), an examination of the CIs provides a more accurate assessment of the size of the effects in the context of a generalized linear mixed model. The estimate values in Table 2 revealed the presence of a 95% chance that the calculated confidence intervals contain the true population parameters. Overall, the effects appear to be strong, but some CIs are significantly wider than others. Unlike the other fixed effects, the Position effect is practically nearing zero providing us enough certainty to believe that this effect is weak along with its interaction with Language.

**Table 2:** Summary of the optimal GLME model and its parameter estimates.

**Effects**	**Estimate**	**SE**	***z*-value**	**Pr(>|z|)**	**CI-lower**	**CI-upper**	**δ^a^**
Intercept	−0.21	0.59	−0.36	0.723	−1.37	0.95	
Language-English	1.15	0.75	1.53	0.126	−0.32	2.62	0.002
Context-pseudo-word	3.51***	0.88	4.01	0.000	1.79	5.22	0.014
Context-word	5.18***	0.79	6.55	0.000	3.63	6.72	0.031
Position	0.25	0.17	1.48	0.139	−0.08	0.59	0.002
Language-English: context-pseudo-word	−3.38***	1.09	−3.10	0.002	−5.51	−1.24	0.007
Language-English: context-word	−4.12***	1.03	−4.00	0.000	−6.14	−2.10	0.010
Language-English: position	−0.47	0.22	−2.15	0.032	−0.89	−0.04	0.003
Context-pseudo-word: position	−0.72***	0.25	−2.85	0.004	−1.22	−0.23	0.007
Context-word: position	−0.98***	0.21	−4.58	0.000	−1.40	−0.56	0.013
Language-English: context-pseudo-word: position	0.98***	0.32	3.11	0.002	0.36	1.60	0.007
Language-English: context-word: position	1.20***	0.29	4.17	0.000	0.64	1.77	0.010

All effects are estimated with respect to the base levels of each factor which is Arabic for Language and non-word for the Context. Position is treated as a continuous factor.

a For GLMM models, a common procedure used to estimate the effect size is to calculate the marginal (variance explained by fixed effects) and conditional (variance explained by both fixed and random effects) Pseudo R-squared values as recommended by Johnson (2014) based on the “theoretical” method for the specific case of binomial family models such as used in our study. Moreover, we calculated the semi-partial R2 statistic for fixed (population) effects in the GLMM by utilizing the penalized quasi-likelihood estimation method based on linearization as recommended by Jaeger et al. (2017). Significant effect is indicated by asterisk (***p < 0.001).

### Assessing the word superiority effect

**Figure 4:**
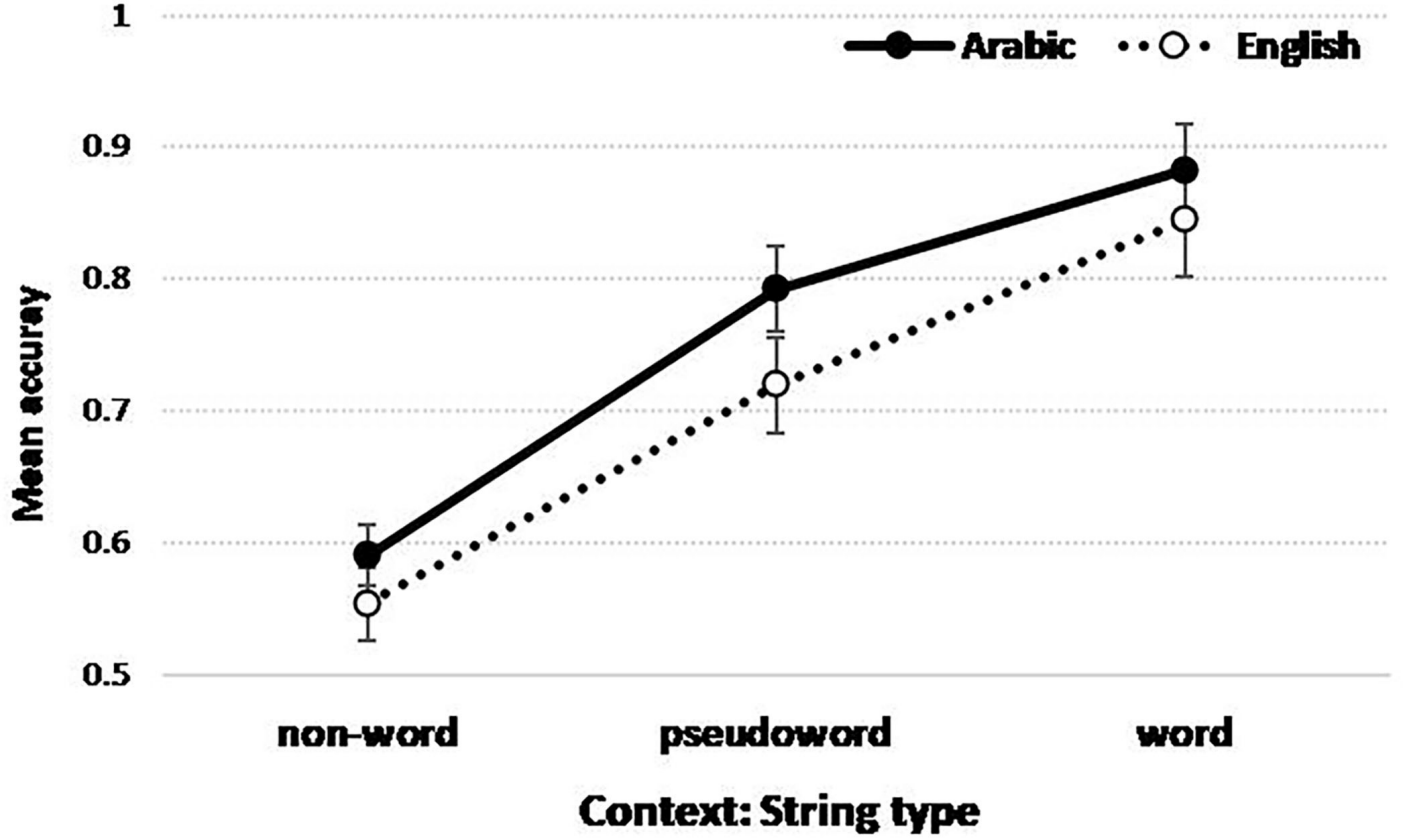
Figure illustrating the interaction in accuracy scores (probabilities) between String type and Language. Mean with +/- standard errors.

The interaction plot between Language and String type (Context) is illustrated in Figure 4.

Figure 4 shows that the accuracy in detecting within-string letter targets is overall higher when the letter target is embedded in words relative to pseudo-words and non-words. This finding confirmed the presence of the word superiority effect regardless of the language. The results also revealed that, compared to the non-word context, word and pseudo-word contexts help letter identification more in Arabic. Moreover, the word vs. pseudo-word difference is smaller in Arabic compared to English.

A better understanding of this pattern can be achieved using multiple comparisons with the false discovery rate (FDR) correction to adjust the *p*-values (see Rouam, 2013). The results showed that for Arabic, the comparison between word and pseudo-word contexts is marginally significant (*p* = 0.0633) and between pseudo-word and non-word contexts is highly significant (*p* = 0.0019). For English, the comparisons between word and pseudo-word contexts (*p* = 0.0175) and between the pseudo-word and non-word contexts (*p* = 0.0058) are significant. Moreover, by comparing both languages, the higher accuracy in Arabic is marginally significant for word (*p* = 0.0906) and pseudo-word (*p* = 0.0965) contexts relative to English but no significant difference between both languages in the non-word context (*p* = 0.5414).

**Figure 5:**
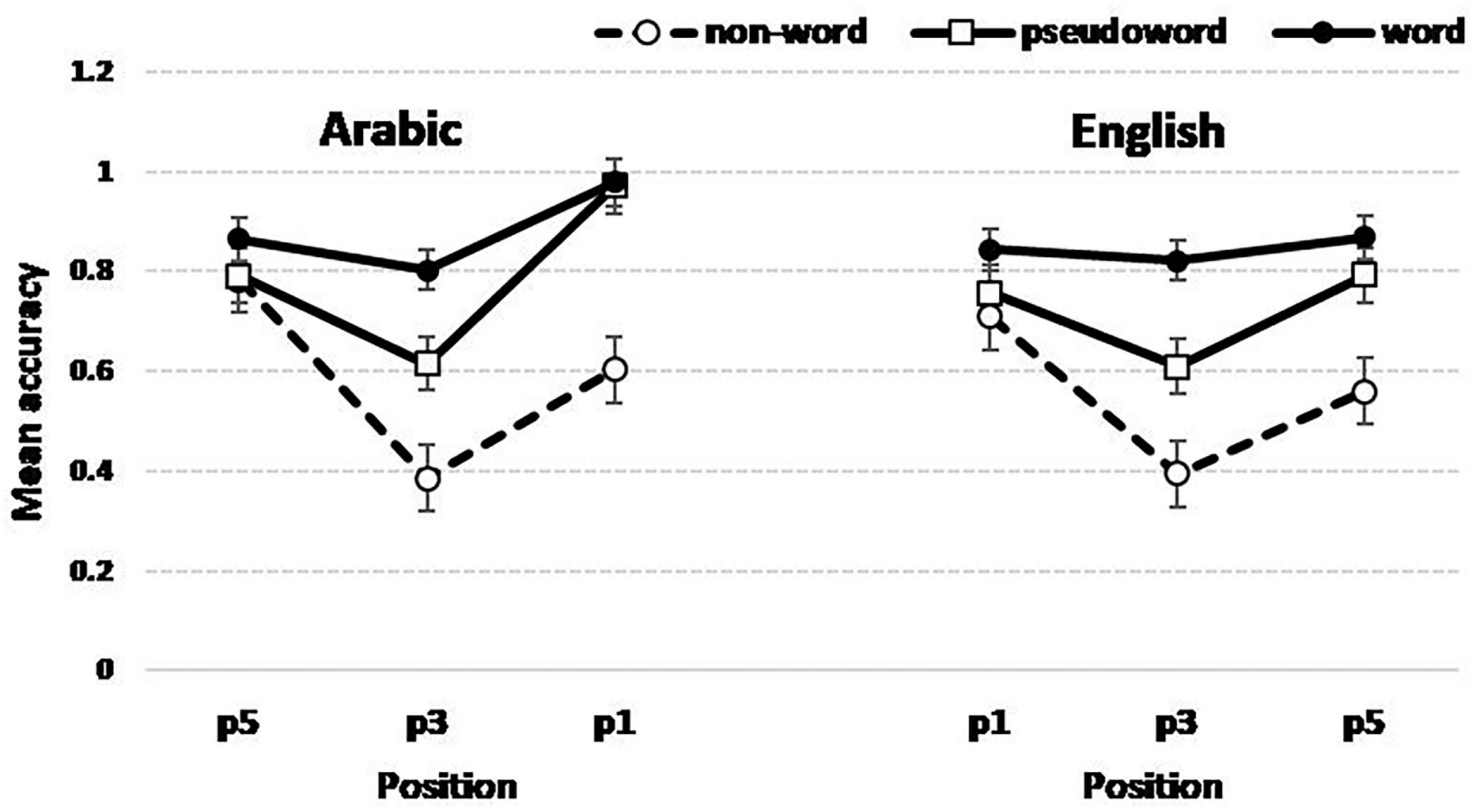
Accuracy as a function of Language, String type (word, pseudo-word, and non-word), and letter Position. Note that the layout of the letter position in the left panel is from right to left in line with the reading direction in Arabic.

Figure 5 shows the triple interaction plot between all the fixed factors in the design. It is therefore important to examine and compare the interaction between the String type and Position in each language.

In English, the results showed a first-letter advantage (the difference between the accuracy in p1 and p3) only for non-words (*Z*-ratio = 3.35, *p* = 0.003) and a marginal advantage for pseudo-words (*Z*-ratio = 1.83, *p* = 0.0911). In Arabic, the first-letter advantage is present in all three contexts: words (*Z*-ratio = 5.27, *p* < 0.0001), pseudo-words (*Z*-ratio = 5.45, *p* < 0.0001), and non-words (*Z*-ratio = 2.38, *p* = 0.0429).

In English, an alphabetic language reads from left to right, and our bilingual participants tend to grab primarily the first letter as key information in such noisy contexts as non-words. In Arabic, this difference weakened progressively as we move from words to non-words which point toward the key role of morphological structure, and the consonantal root, in particular, in spawning top-down influence to detect within-string letters. Indeed, the non-word context is highly noisy since non-words show no recognizable root material.

To understand what is driving the three-way interaction illustrated in Figure 5, we run partial interactions testing Position X Language for each context. The results revealed the absence of this interaction for non-words (*p* = 0.0632) and its presence for words (*p* = 0.0129) and pseudo-words (*p* = 0.0255).

**Figure 6:**
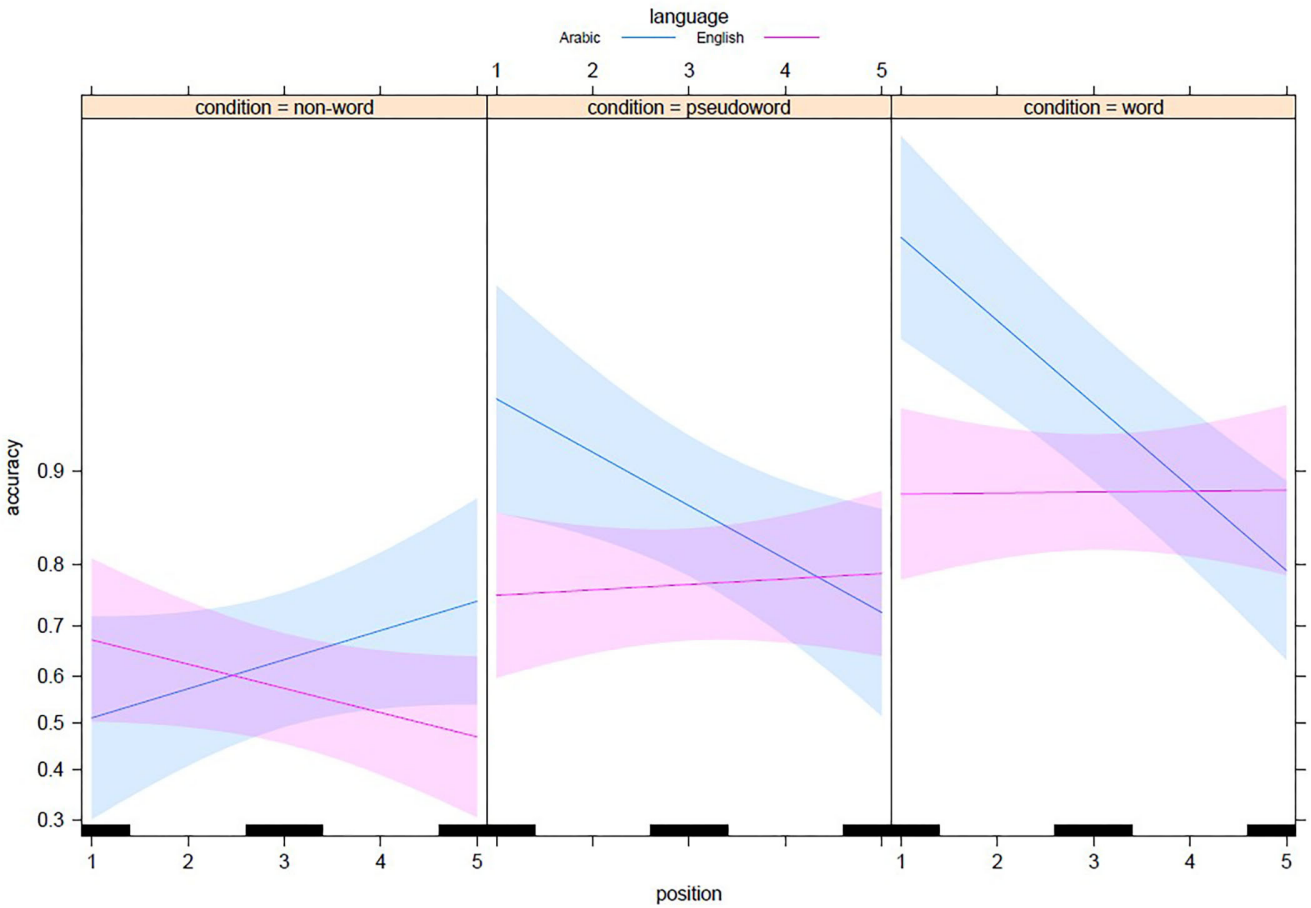
Visualization of the GLME model predictions for the word superiority effect in Arabic and English as a function of String type and Position. The bands correspond to the confidence interval for each regression line.

Figure 5 illustrates the way the optimal GLME model predicts the word superiority effect in both languages. Therefore, we can notice from the predictions of the slopes that the non-word strings represent the most challenging context for Arabic readers where they succeeded in English better than in their native language. The model predictions plotted in Figure 6 confirmed that the fragility of the accuracy in the non-word context lends itself to the above explanation in terms of the saliency of the root in cementing units or chunks of letters to facilitate the letter-in-string identification and modulate the shape of the serial position function in this paradigm.

## General discussion

In this paper, we set out to investigate the word superiority effect in Arabic. We capitalized on a unique feature of Arabic language, namely the role of prosodic structure in defining word likeness, to create more stringent stimuli than used by Jordan et al. (2010). We also included both Arabic and English with bilingual readers with the aim to establish a baseline condition that would allow us to assess the magnitude of visual crowding and visual acuity when comparing their performance toward the same types of stimuli in both languages. Moreover, we capitalized on the close relationship between the morphological structure of a word and its visual form in Arabic to explore the potential interaction between the two. We specifically examined whether the serial position factor would modulate the word superiority effect in Arabic by comparing the letter identification accuracy in the first, the middle, and the final positions.

Our results established the presence of the word superiority effect in Arabic, which constitutes an important step toward the cross-linguistic and cross-script validation of the word superiority effect. We extend previous findings in languages using Roman alphabet (e.g., Baron, 1978; Lukatela et al., 1981; Grossi et al., 2009) and Chinese (Chen et al., 2018) to yet another typologically distant language, Arabic. Taking advantage of the idiosyncratic morphophonological and orthographic systems of Arabic, we thus confirm the robustness of the word superiority effect across languages and conditions (Spector and Purcell, 1977; Peterzell et al., 1990). As a matter of fact, Arabic and English show significantly different word structures and orthographic properties that should highlight the exact nature of the effect of morphology and orthography on the word superiority effect.

Our findings showed that we obtained the word superiority effect in both Arabic and English. Participants were more accurate at identifying the target letter in words than in pseudo-words and non-words regardless of the language. This is then clear additional cross-linguistic evidence for the word superiority effect. Another element of cross-linguistic validation relates to the presence in Arabic of the pseudo-word superiority effect that is believed to be explained by the partial activation of shared information between the real word and their neighbors that results in a reinforcement mechanism of their component letter activation (McClelland and Rumelhart, 1981; Tainturier, 2013). However, the idiosyncratic aspect of pseudo-words in Arabic, namely that they share the same prosodic structure (word pattern) as real words, is likely to explain the special status of pseudo-words in Arabic compared to their counterparts in English.

We hypothesized that pseudo-words should pattern with words in Arabic much more than they would do in English. This follows from the fact that in Arabic, pseudo-words are specifically built on the same word patterns as words, which increase the likelihood of their word likeness, their Gestalt, and pronounceability (e.g., Baron and Thurston, 1973). The results of this study show that, unlike in English, words and pseudo-words in Arabic seem to facilitate letter identification to the same extent. We argue that this is due to the salience of the word template in Arabic, which provides a new type of evidence for the central role of the template in Arabic lexical representations. Once presented with a pseudo-word, our participants tend to extract the word pattern, which leaves any consonantal material to be treated as a root. The robust interaction between Language, Context, and Position of the letter revealed that the specificities of orthography modulate the shape of the serial position function when participants perform a letter-in-string identification task. These results suggest that the pseudo-word superiority effect in Arabic may be subtended by regularities operating at the level of sublexical orthomorphological representations (Grainger et al., 2003).

It has been argued that the pseudo-word superiority effect may be more salient in languages with shallow or regular orthography [see Coch and Mitra's (2010) and Ripamonti et al. (2018) ERP data]. The fact that Arabic pseudo-words patterned more with words than they did in English points toward a possible effect of orthographic shallowness/opacity on the word superiority effect.

There is yet another interesting pattern in our results that can be attributed to the differential role of prosodic structure in the representation and processing of words between Arabic and English. In English, the results show that the first-letter advantage was significant only in the context of non-words. More precisely, the accuracy with which the first letter was identified is significantly higher compared to the foveal (medial) letter. In Arabic, the first-letter advantage is observed in all three contexts (word, pseudo-word, and non-word), but this advantage tends to wear off in the non-word context. This could be attributed to the fact that Arabic words and pseudo-words in this study share the same word pattern. Therefore, unlike in non-words, once the word pattern is extracted during the letter identification task, the remaining material is likely to be a root material (McCarthy, 1979, 1981; Prunet et al., 2000; Boudelaa and Marslen-Wilson, 2004; Idrissi and Kehayia, 2004; Prunet, 2006; Idrissi et al., 2008; Idrissi, 2018). This may explain why letter identification is less straightforward in non-words since the non-word context does not allow such a straightforward word decomposition that allows access to these abstract units (i.e., word pattern and root). Non-words do not maintain the same prosodic structure as words. In conclusion, regardless of the language, non-words trigger the same strategy: letter-by-letter parsing.

In short, the results suggest the additional role of morphophonological processing in word reading Arabic (in addition to orthographic processing).

Although only the first, the middle, and the last positions were analyzed in this study, the results relative to non-words in both languages may strongly suggest the presence of the W-shaped curve previously reported in many studies using the Latin alphabet (e.g., Tydgat and Grainger, 2009; Marzouki and Grainger, 2014). Thus, the pattern of the W-shaped curve seems to be language-independent when we manipulate noisy context—completely random letter strings (see also for Arabic letters and digits, Marzouki et al., 2016). In the presence of the non-word context, low-level visual mechanisms specifically visual crowding and visual acuity intervene predominantly (see Figure 1). The more we shift to the pseudo-word context, the more we see abstract units (visual word form for English and word pattern in Arabic) intervene.

The across-the-board first-letter advantage observed in English may suggest that reading the Latin alphabet deploys a linear and more phonologically but less morphologically informed mechanism. In Arabic, unlike in English, the first-letter advantage is weaker in the context of non-words. This may be due to the fact that successful word reading, that is, successful grapheme recognition, may be guided by morphological structure much more than it is in English. That this advantage was weaker in non-words in Arabic may suggest that the processing mechanisms underlying grapheme recognition fail when proper morphological analysis of the stimulus fails or is slowed down due to the illegal nature of the consonants and prosodic structure. This points toward the central role of phonological and orthographic processing in letter identification (Saito and Masuda, 2000, for Japanese; Ziegler et al., 1997). This can be taken as evidence for top-down modulation during visual word identification in Arabic. Indeed, Heilbron et al. (2020) found that, unlike non-word contexts, word contexts enhance individual letter representations in early visual cortex when the participants perform an orthographic discrimination task. Heilbron et al. (2020) noticed an increase in brain activity within areas typically associated with the reading network and the processing of individual letter information in the visual cortex.

## Data availability statement

The original contributions presented in the study are publicly available. This data can be found here: https://osf.io/x6gb8/.

## Ethics statement

The studies involving human participants were reviewed and approved by Qatar University Institutional Review Board. The patients/participants provided their written informed consent to participate in this study.

## Author contributions

YM designed the study and performed the statistical analysis. SA-O, MA-T, and AI contributed to the conception and the design of the stimuli. SA-O collected the data. YM and AI discussed and interpreted the results and wrote the manuscript. All authors read and approved the submitted version.

## Funding

Open Access funding provided by the Qatar National Library.

## Conflict of interest

The authors declare that the research was conducted in the absence of any commercial or financial relationships that could be construed as a potential conflict of interest.

## Publisher's note

All claims expressed in this article are solely those of the authors and do not necessarily represent those of their affiliated organizations, or those of the publisher, the editors and the reviewers. Any product that may be evaluated in this article, or claim that may be made by its manufacturer, is not guaranteed or endorsed by the publisher.
