# Supplementary material for: Can the word superiority effect be modulated by serial position and prosodic structure?
Source: Front Psychol. 2022 Aug 5;13:915666. doi: 10.3389/fpsyg.2022.915666 (PMC9389116; doi:10.3389/fpsyg.2022.915666)
Supplement: Supplementary file 1 [file Data_Sheet_1.PDF]

Appendix

Arabic Stimuli List (Database: Aralex)

Words	IPA	Orth. Frequency	Target p1	pseudo1	Target p2	pseudo3	Target p5	pseudo5
عملية	[ʕmɫjhun]	611.23	ع	عبلية	ل	عجلية	ة	عملنة
دراسة	[drʔshun]	119.98	د	دطاسة	ا	دكاسة	ة	درابة
توقيع	[twqjʕun]	100.86	ت	تسقيع	ق	تضقيع	ع	توقتع
تتدخل	[ttdXɫ]	100.65	ت	تبدخل	د	تبدخل	ل	تتدطل
تراجع	[trʔʤʕun]	83.12	ت	تساجع	ا	تشاجع	ع	ترامع
مواقف	[mwʔqfun]	76.96	م	مشاقف	ا	مخاقف	ف	مواثف
مواقع	[mwʔqʕun]	68.82	م	مجاقع	ا	مساقع	ع	مواصع
مفهوم	[mfhwmun]	67.7	م	مصهوم	ه	مخهوم	م	مفهزم
الذهب	[ʔɫðhb]	47.36	ا	اجذهب	ذ	اكذهب	ب	الذسب
تقدير	[tqdjrun]	44.73	ت	تحدير	د	تضدير	ر	تقدثر
عوامل	[ʕwʔmɫun]	43.2	ع	علامل	ا	عمامل	ل	عواصل
يطالب	[jtˤʔɫb]	41.43	ي	يهالب	ا	يصالب	ب	يطانب
الحدث	[ʔɫħdθ]	40.76	ا	ازحدث	ح	ابحدث	ث	الحبث
مطلعة	[mtˤɫʕtun]	39.82	م	مصلعة	ل	منلعة	ة	مطلفة
موجود	[mwʤwdun]	26.5	م	مزجود	ج	معجود	د	موجزد
حديثة	[ħdjθtun]	29.29	ح	حطيثة	ي	حجيثة	ة	حديبة
مكتبة	[mktbtun]	26.5	م	مهتبة	ت	مجتبة	ة	مكتنة
تفاهم	[tfʔhmun]	23.33	ت	تضاهم	ا	تناهم	م	تفاطم
حملات	[ħmɫʔtun]	22.81	ح	حقلات	ل	حضلات	ت	حملوت
جلسات	[ʤɫsʔtun]	22.81	ج	جاسات	س	جبسات	ت	جلسوت
ذهبية	[ðhbjtun]	22.08	ذ	ذوبية	ب	ذلبية	ة	ذهبنة
مدارس	[mdʔrsun]	21.9	م	مجارس	ا	مكارس	س	مداجس
مطلوب	[mtˤɫwbun]	20.7	م	مجلوب	ل	مذلوب	ب	مطليب
تعامل	[tʕʔmɫun]	19.77	ت	تذامل	ا	تصامل	ل	تعاخل
ناجحة	[nʔʤħtun]	18.36	ن	نطجحة	ج	نمجحة	ة	ناجثة

حادثة	[ħʔdθtun]	13.65	ح	حلدثة	د	حصدثة	ة	حادنة
تركيب	[tɾkjbun]	12.93	ت	تذكيب	ك	تزكيب	ب	تركنب
خارجي	[Xʔɾʤjun]	12.87	خ	خزرجي	ر	خبرجي	ي	خارمي
مجلسي	[mʤɫsj]	12.51	م	مخلسي	ل	محلسي	ي	مجلني
مقدار	[mqdʔɾun]	12.12	م	مضدار	د	مشدار	ر	مقدنر
محتمل	[mħtmɫun]	10.95	م	مضتمل	ت	محتكل	ل	محتطل
توقيف	[Twqjfun]	10.95	ت	تنقيف	ق	توقبف	ف	توقنف
دوافع	[dwʔfʕun]	10.59	د	درافع	ا	دواجع	ع	دواذع
متصلة	[mtsˤɫtun]	10.38	م	معصلة	ص	متصبة	ة	متصنة
تحميل	[tħmjɫun]	9	ت	تصميل	م	تحمضل	ل	تحمطل
تنازل	[tnʔzɫun]	8.97	ت	تجازل	ا	تنابل	ل	تنائل
توصيل	[twsˤjɫun]	8.53	ت	تجصيل	ص	توصخل	ل	توصجل
مدافع	[mdʔfʕun]	8.14	م	مجافع	ا	مدالع	ع	مداكع
متوقع	[mtwqʕun]	7.98	م	مصوقع	و	متوثع	ع	متوصع
مكتوب	[mktwbun]	7.85	م	مجتوب	ت	مكتلب	ب	مكتيب
مركبة	[mɾkbtun]	7.15	م	مزكبة	ك	مركجة	ة	مركخة
مدخلا	[mdXɫʔ]	6.89	م	مزخلا	خ	مدخصا	ا	مدخطا
تدريس	[tdɾjsun]	6.27	ت	تبريس	ر	تدرجس	س	تدرطس
مقدرة	[mqdɾtun]	5.54	م	مفدرة	د	مقدبة	ة	مقدنة
منزلي	[mnzɫj]	5.46	م	مفزلي	ز	منزشي	ي	منزري
كتابي	[ktʔbj]	5.44	ك	كضابي	ا	كتاقي	ي	كتاجي
منزلة	[mnzɫtun]	5.2	م	مشزلة	ز	منزجة	ة	منزكة
مخرجا	[mXɾʤʔ]	4.84	م	مقرجا	ر	مخرذا	ا	مخرظا
ناجحا	[nʔʤħʔ]	4.76	ن	نطجحا	ج	ناجخا	ا	ناجغا
طالبة	[tˤʔɫbtun]	4.55	ط	طرلبة	ل	طالخة	ة	طالية
لاعبة	[ɫʔʕbtun]	4.45	ل	لوعبة	ع	لاعصة	ة	لاعجة
تداخل	[tdʔXɫun]	4.42	ت	تجاخل	ا	تدانل	ل	تدابل
خروجا	[Xɾwʤʔ]	4.08	خ	خبوجا	و	خروقا	ا	خروزا
تلاعب	[tɫʔʕbun]	3.23	ت	تظاعب	ا	تجاعب	ب	تلاذب
يراجع	[jɾʔʤʕun]	3.04	ي	يثاجع	ا	يراصع	ع	يراسع

سقوطه	[sqwtˤh]	2.99	س	سنوطة	و	سقوظة	ه	سقوجة
تواجد	[twʔʤdun]	2.96	ت	تحاجد	ا	تواضد	د	توابد
مطالع	[mtˤʔɨʕun]	2.26	م	مصالع	ا	مطافع	ع	مطانع
واقفة	[wʔqftun]	2.24	و	واقذة	ق	واقجة	ة	واقبة
أسقطت	[ʔsqtˤt]	2.24	أ	أجقطت	ق	أسقنت	ت	أسقبت

English stimuli list Database: (Balota, 2002)

words	Orth. Frequency	Target p1	pseudo1	Target p3	pseudo3	Target p5	pseudo5
order	12.17	o	ovder	d	omder	r	ordmr
power	12.14	p	pkwer	w	pvwer	r	powvr
start	12.06	s	snart	a	spart	t	stamt
above	12.09	a	acove	o	amove	e	abore
until	12.05	u	ultil	t	uptil	l	untal
drive	12.05	d	dsive	i	dpive	e	drime
black	11.99	b	bnack	a	brack	k	blask
white	11.92	w	wcite	i	wmite	e	whipe
price	11.94	p	pmice	i	paice	e	prine
quite	11.95	q	qoite	i	qmite	e	quine
phone	11.86	p	plone	o	pgone	e	phole
stuff	11.85	s	sbuff	u	snuff	f	stulf
large	11.81	l	lnrge	r	lorge	e	larne
music	11.81	m	mnsic	s	mpsic	c	musnc
level	11.78	l	lavel	v	lnvel	l	levol
sound	11.71	s	spund	u	sgund	d	sourd
issue	11.68	i	insue	s	iosue	e	issve
space	11.64	s	slace	a	snace	e	spave
video	11.61	v	vmdeo	d	vldeo	o	vidno
women	11.62	w	wlmen	m	wnmen	n	womon
water	11.57	w	wmter	t	wlter	r	watgr
today	11.55	t	trday	d	tmday	y	todny
house	11.55	h	hnuse	u	hruse	e	houme
story	11.48	s	smory	o	spory	y	stogy
light	11.48	l	lrght	g	loght	t	ligmt

night	11.49	n	noght	g	naght	t	nigrt
offer	11.5	o	onfer	f	opfer	r	offkr
speed	11.42	s	sgeed	e	sreed	d	speod
green	11.42	g	gpeen	e	gmeen	n	greon
voice	11.31	v	vnice	i	vlice	e	voire
title	11.29	t	tntle	t	titne	e	titme
solid	10.1	s	snlid	l	solnd	d	solsd
scale	10.11	s	snale	a	scane	e	scave
clock	10.11	c	cnock	o	clomk	k	clopk
stick	10.25	s	snick	i	stimk	k	stiok
limit	10.35	l	lnmit	m	limot	t	limat
frame	10.36	f	fname	a	frane	e	frale
dream	10.39	d	dneam	e	dresm	m	drelm
stone	10.4	s	snone	o	stoze	e	stoge
mouth	10.44	m	mnuth	u	mourh	h	mounh
audio	10.44	a	andio	d	audno	o	ardlo
topic	10.45	t	tnpic	p	topnc	c	topsc
floor	10.48	f	fnoor	o	flonr	r	flozr
funny	10.51	f	fjnny	n	funpy	y	fungy
mouse	10.54	m	mnuse	u	moune	e	mouye
peace	10.61	p	pnace	a	peane	e	peade
brown	10.65	b	bnown	o	broyn	n	broln
style	10.67	s	snyle	y	styne	e	styqe
heart	10.73	h	hnart	a	heant	t	heazt
break	10.77	b	bneak	e	brenk	k	brelk
magic	10.78	m	mngic	g	magnc	c	magsc
piece	10.74	p	pnece	e	piene	e	piele
final	10.8	f	fbnal	n	finvl	l	finpl
store	10.88	s	slore	o	stofe	e	stome
brain	10.9	b	bnain	a	bramn	n	braln
table	10.93	t	tnble	b	tabne	e	taboe
watch	10.95	w	wntch	t	watsh	h	watph
media	10.97	m	mndia	d	medna	a	medma
movie	11.02	m	mnvie	v	movre	e	movge
color	11.22	c	cnlor	l	colpr	r	colmr
